# Climatic and Altitudinal Influences on Variation in *Macaca* Limb Morphology

**DOI:** 10.1155/2011/714624

**Published:** 2011-10-18

**Authors:** Karen J. Weinstein

**Affiliations:** Department of Anthropology, Dickinson College, P.O. Box 1773, Carlisle, PA 17013, USA

## Abstract

This study compares limb lengths and joint diameters in the skeletons of six macaque species (*Macaca assamensis*, *M. fascicularis*, *M. fuscata*, *M. mulatta*, *M. nemestrina*, and *M. thibetana*) from a broad range of habitats and climates in order to test whether ambient temperatures, latitude, and altitude influence interspecific variation in limb morphology in this widely dispersed genus. Analysis of variance, principal component analysis, and partial correlation analysis reveal that species from temperate latitudes and high elevations tend to have short limbs and large joint diameters for their sizes while species from tropical latitudes and low elevations tend to have long limbs and small joint diameters. Interspecific variations in intra- and interlimb length proportions also reflect phylogeny and subtle differences in locomotion. The results of this study suggest that climatic conditions are important factors among many ecological variables that influence limb morphology in this geographically widespread genus.

## 1. Introduction

Ecogeographic patterns, such as Bergmann's and Allen's rules, have long been of interest to evolutionary biologists testing hypotheses about the evolution of geographic variation within and among closely related species. These ecogeographic rules, which state that body mass and appendage lengths vary by climate and latitude among geographically dispersed endothermic species in response to thermoregulation, have been invoked to explain variation in body size and proportions in many mammals, birds, and other vertebrate species [4–10]. Among primates, ecogeographic patterns have been used to explain variation in body size in lemurs [11] and baboons [12–14], cranial size, body size, and relative tail length in macaques [15–20], and body size and proportions in modern humans [21–27] and Pleistocene hominins [28–34]. 

**Figure 1:**
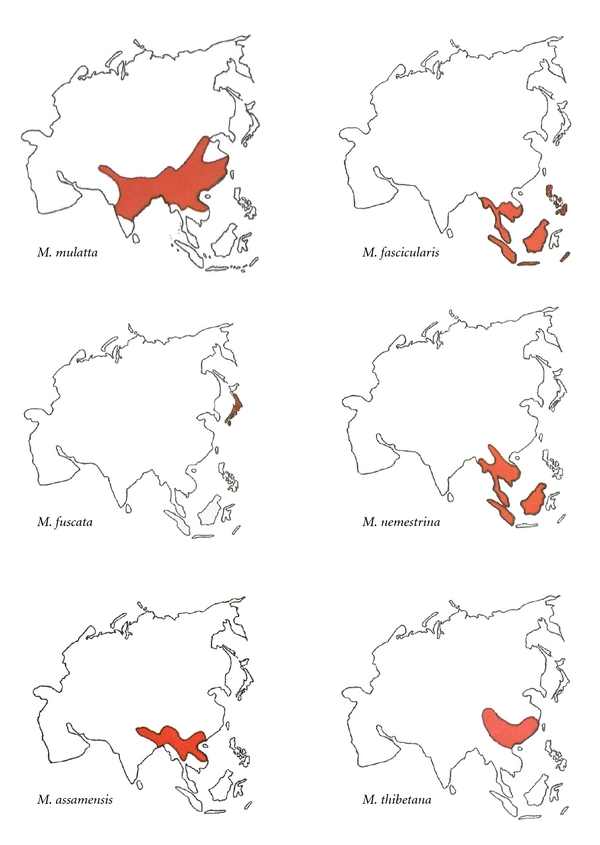
Geographic ranges of *Macaca* species used in this study.

*Macaca* is a particularly useful genus for modeling the significance of geographic dispersal and ecological variation in the evolution of living and fossil primates. Macaque species inhabit the greatest geographic range of any nonhuman primate genus from 10° South to over 40° North latitude, habitats that extend from lowland tropical forests to temperate climates and altitudes in excess of 2500 m, and stretching from Afghanistan eastward through Taiwan and Japan, and a single species in Northwest Africa (Figure 1). Despite this geographic diversity, *Macaca* is a monophyletic genus comprised of 22 species that Fooden [1, 35] and Delson [2] divide into four species groups based on male reproductive anatomy and which Groves [3] divides slightly differently into six species groups (Table 1). Fossil and biomolecular data indicate that the earliest macaques emerged at circa 7 mya in Northern Africa [1–3]. Subsequent speciation and dispersal occurred first in Fooden's *sylvanus-silenus* species groups, including the broad geogeographic range of pig-tailed macaques (*M. nemestrina*), followed by diversification within the *fascicularis* and *sinica* species groups during the late Pliocene through Pleistocene epochs [1, 2]. Fooden [1] demonstrates that, while there is a great deal of sympatry among macaques, species within each species group are allopatric. Within Fooden's *fascicularis* species group, crab-eating macaques (*M. fascicularis*) are the most primitive species with other members of this species group, rhesus (*M. mulatta*), Japanese (*M. fuscata*), and Taiwanese macaques (*M. cyclopis*), as descendant populations continuously distributed throughout Southeast and mainland Asia into Japan and Taiwan [1, 2]. Central to this study, an early form of rhesus macaques diverged from crab-eating macaques in the northern part of its range, spread northward and eastward to India and China and subsequently divided into eastern and western subspecies [36, 37]. Japanese macaques then diverged from rhesus macaques and dispersed to the Japanese archipelago [38]. Fooden [1] argues that the species divisions between crab-eating macaques and rhesus macaques and between rhesus macaques and Japanese macaques are somewhat arbitrary implying introgression between species within the *fascicularis* species groups, an observation supported by genetic evidence [39]. Within the *sinica* species group, Tibetan (*M. thibetana*) and Assamese macaques (*M. assamensis*) are closely related allopatric species that extend from tropical latitudes (Assamese macaques) to northern, temperate regions (Tibetan macaques) [1, 2]. Molecular data largely support Fooden's and Delson's morphologically-based phylogenetic scenarios [38–44]. These data not only support the monophyletic origins of the *Macaca* genus [39] but also indicate the diverse intraspecific variation within rhesus macaques [41] and paraphyletic relationships in species within the *fascicularis* species group in general [38, 39]. Similarly, the broad geographic range of pig-tailed macaques (*M. nemestrina*) within the *silenus* species group translates into a great intraspecific genetic diversity [43].

Morphological variation within and among macaque species tends to conform to ecogeographic patterns. In Japanese macaques, body weight and trunk length increase with decreasing winter temperatures [45–47]. Skull size increases with increasing latitude within the *silenus* and *fascicularis* species groups [15, 16]. Relative tail length decreases with increasing latitude within all macaques and especially within the *fascicularis* species group [1, 17, 19]. Fooden [1] explains that an elongated tail is the primitive condition as the genus first evolved in tropical latitudes and subsequently reduced in length convergently within each species group as populations dispersed northward. Short hindlimbs in Japanese and Tibetan macaques compared with other cercopithecines, moreover, may have developed in response to the cold temperatures and high-altitude habitats of these two species [48]. Variation in limb proportions within *Macaca*, however, has yet to be examined for ecogeographic patterning despite the fact that limb proportions are greatly affected by long-term climatic conditions. It is important to note, moreover, that while Bergmann's and Allen's rules have typically been applied to intraspecific variation in a wide variety of mammalian species, previous studies of morphological variation in skull size, body size, and tail length in macaques [1, 17, 19] have identified ecogeographic patterns interspecifically within a species group. Given the morphological ambiguity in distinguishing species divisions within the four species groups [1–3, 49] and the documented introgression in closely related species based on molecular studies [39], it seems reasonable to test for ecogeographic patterning interspecifically in closely related macaque species.

This study examines whether altitude and latitude and ambient temperature influence interspecific variation in *Macaca* limb proportions by comparing the skeletons of Assamese (*Macaca assamensis*), crab-eating (*M. fascicularis*), Japanese (*M. fuscata*), rhesus (*M. mulatta*), pig-tailed (*M. nemestrina*), and Tibetan macaques (*M. thibetana*) (Figure 1). These six species vary in ecogeography, body size, and degree of arboreal or terrestrial quadrupedal locomotion and represent species within three of Fooden's [1] and Delson's [2] four macaque species groups. Within the *fascicularis* species group, rhesus macaques, a mostly terrestrial and small-bodied species, thrive in degraded forests at the edge of human habitation from Afghanistan eastward through China and from tropical latitudes in Southeast Asia northward to above 35° North latitude including elevations over 3000 m [50–55]. Crab-eating macaques, a small-bodied, predominantly arboreal species also in the *fascicularis* species group, inhabit lowland secondary riverine forests in the Philippine and Indonesian archipelagos and Southeast Asia [56–58]. Japanese macaques, a larger semiterrestrial species in the *fascicularis* species group, inhabit latitudes of 31–41° north in Japan [47]. Within the *sinica* species group, the large-bodied, predominantly arboreal Assamese macaques inhabit broadleaf evergreen forests at elevations of 150–2750 m in Southeast Asia [59]. Closely related Tibetan macaques, a large-bodied terrestrial species, are restricted to East Central China at 25–33° North latitude at elevations of 1000–2400 m [60, 61]. Pig-tailed macaques, a large-bodied terrestrial species in the *silenus* species group, inhabit lowland primary forests in Western Thailand, Malaysia, and Sumatra [56, 57, 62].

This study compares *Macaca* interspecific variation in limb proportions and joint diameters to test for the affects of climatic conditions in this ecogeographically diverse genus. While interspecific variation in limb morphology is influenced by many factors, including differences in body size, locomotion, and phylogeny, climatic conditions, such as altitude, latitude, and ambient temperatures, also should influence the degree of interspecific variation in limb proportions in closely related species within this genus. Those species from the highest latitudes and altitudes (Tibetan, rhesus, and Japanese macaques) ought to exhibit short fore- and hindlimbs, smaller limb proportions for their size, and larger joint diameters compared with their congeners from tropical, lowland environments (crab-eating, Assamese, and pig-tailed macaques).

## 2. Materials and Methods

### 2.1. Macaque Skeletons, Osteometric Variables, and Climate

I compare limb lengths and joint diameters to estimate of overall size in the skeletons of six species of macaques that were collected from their natural habitats (Table 2). All individuals are adults based on long bone epiphyseal fusion and the presence of the maxillary third molars in occlusion. I measured maximum lengths of the humerus (HUM), radius (RAD), femur (FEM), and tibia (TIB) to the nearest 0.5 mm with an osteometric board. Maximum lengths of the forelimb (FORE) and hindlimb (HIND) are the sum of HUM and RAD and FEM and TIB, respectively. Given that articular surfaces of weight-bearing limb bones reflect both body mass and locomotor behavior [63] and that macaques vary in the degree to which each species engages in arboreal and terrestrial quadrupedal locomotion, I also measured the anteroposterior diameters of the humeral (HHAP) and femoral heads (FHAP) to the nearest 0.1 mm with Mitutoyo's digital sliding calipers. 

In order to compare interspecific variation in limb proportions, I employ two independent methods for estimating overall size. First, I estimate body mass (M) for each individual using a regression equation of the supero-inferior diameter of the femoral head (FHSI) and body weight in cercopithecines ([64]; Table 3). This equation, Log  M  (kg) = [2.389 × Log  FHSI  (mm)] − 4.451, is based on the relationship between FHSI and known body mass in cercopithecine species and predicts to within a 13% range of actual body weight in individual monkeys with known body weights. In order to adjust for detransformation bias when converting log-transformed values back to their original units, I use the quasi-maximum likelihood estimator correction factor, which represents the product of the dependent variable with EXP(*s* ^2^/2) where *s* ^2^ represents the residual mean square error of the equation in logarithmic units and is a commonly used correction factor for detransformation bias in morphometric studies [64, 65]. I also use the geometric mean (GM) measured on each individual as a second method for estimating overall size [66]. Interspecific variation in overall size does not differ in pattern or scope when using M or GM (Table 3).

I traced the latitude and longitude of each monkey's recovery site by recording its location from museum catalogues or collectors' field notes. I then recorded the latitude (LAT), altitude (ALT), and the lowest monthly average temperature (TMIN) from the weather station situated nearest to each recovery site using the Global Climate Categories CD-ROM ([67]; Table 2). Given that *Macaca* first appeared in subtropical latitudes in Northern Africa and subsequently dispersed to temperate latitudes and higher elevations [1–3], cold ambient temperatures are likely to be a strong selective pressure in this genus. Thus, my analysis uses the lowest mean monthly temperature as an important climatic variable to consider in interspecific variation in macaque limb proportions. I calculated TMIN for each weather station by averaging the low temperature for the coldest month for each recorded year.

### 2.2. Statistical Procedures

Intralimb length proportions, which represent the length of the distal limb segment of the fore- or hindlimb relative to the length of its proximal segment, vary with climate. Populations from colder climates tend to have lower intralimb length proportions than their tropical counterparts in accordance with Allen's rule, patterns that are apparent in a wide variety of mammalian species [68]. While the application of Allen's rule has typically been applied to intraspecific variation across a wide range of mammals, previous studies of morphological and ecogeographic variation in *Macaca* have applied Allen's rule to interspecific samples of species within particular species groups [1, 17, 19]. I test whether intralimb length proportions vary with climate in macaque species. Specifically I hypothesize that the values of RAD/HUM and TIB/FEM in Tibetan, rhesus, and Japanese macaques from colder climates and higher altitudes and latitudes should be less compared with crab-eating, pig-tailed, and Assamese macaques from lower elevations and tropical regions. Given that all macaque species engage in some form of arboreal or terrestrial quadrupedalism, the fore- and hindlimbs of these species should be nearly equal in length, and thus, their interlimb length proportions, FORE/HIND, should not vary between species [63]. 

I compare intra- and interlimb length proportions in sex-specific species groups by calculating log-transformed indices of RAD/HUM, TIB/FEM, and FORE/HIND according to the methods described in Ruff [63]. Each index represents the equation of log (*Y*/*X* ^*b*^) where *Y* is the dependent variable in a regression model, *X* is the independent variable, and *b* is the predicted slope given isometric scaling between the independent and dependent variables [63, 69]. While there are many statistical procedures that evaluate skeletal proportions between taxonomic groups in which both the dependent and independent variables are measured with error, log-transformed indices to compare intra- and interlimb length proportions have been a useful method in a variety of studies in humans and nonhuman primates [26, 63]. In log-transformed indices that represent intra- and interlimb length proportions, the predicted isometric slope is 1.0. As in Ruff [63], sex-specific species means of log-transformed indices are compared using ANOVA and the Games-Howell test, a nonparametric post hoc statistical test that is appropriate for comparing samples of unequal size that lack normal variance (Table 4). 

I also compare individual limb lengths and joint diameters with M and GM by calculating log-transformed indices using the methods described above. When M is the independent variable, the predicted isometric slope in the log-transformed index is 0.333, and, when GM is the independent variable, the predicted slope is 1.0. For each comparison, I remove the dependent variable from GM. As in the analyses of intralimb length proportions, I compare sex-specific mean log-transformed indices of each species using ANOVA and the Games-Howell post hoc tests (Tables 5–8). If climate influences interspecific variation in limb proportions, then the highland and temperate-dwelling species ought to have shorter limb lengths and larger joint diameters for their sizes than the lowland and tropical dwelling species. 

Interspecific comparisons of long-bone lengths scaled with body mass in primates and other mammals may contain a phylogenetic signal, the tendency for closely related species to resemble one another compared with more distantly related species due to stochastic evolution (e.g., [70, 71]). When a phylogenetic signal is present, various phylogenetic comparative methods can be used to correct for this phenomenon [70, 71]. While the six species analyzed here differ from one another in their phylogenic relationships (Table 1), testing for the presence of a phylogenetic signal is outside the scope of this study for the following reasons. Most studies that detect a phylogenetic signal in interspecific comparisons of long-bone lengths scaled with body mass in primates and other mammals are based on samples of 20 or more species means compared across high taxonomic levels [70, 71]. It is unclear how strong a phylogenetic signal would be when the interspecific comparisons are of individuals at a lower taxonomic level, such as individuals within a single genus of macaques as examined here. Second, most interspecific comparisons of long-bone lengths scaled to body mass in primates are used to differentiate species across broad locomotor groups, such as differentiating leapers from brachiators from generalized arboreal quadrupeds (e.g., [63]). All of the macaques compared in this study, however, are categorized as general arboreal and terrestrial quadrupeds, with each species varying to some degree in its use of terrestrial and arboreal substrates. It is unclear whether or not a phylogenetic signal can be detected within a single locomotor group and of the genus-level interspecific comparisons of long-bone lengths scaled to body mass as performed here. 

**Figure 2:**
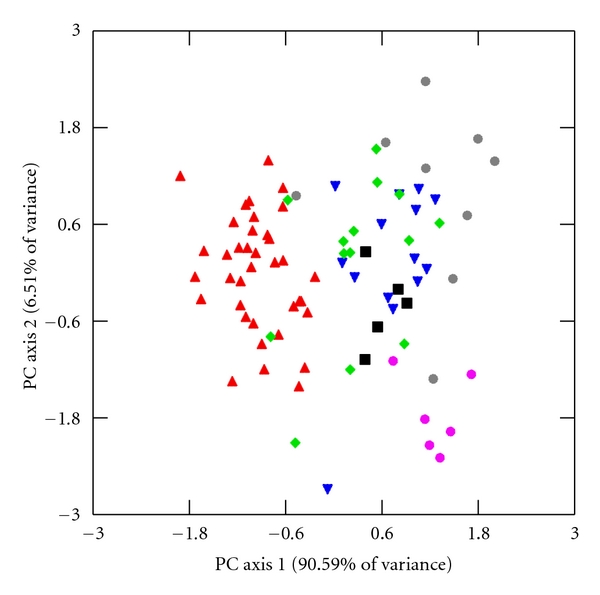
First and second PC factor scores of log-size-and-shape variables in males. The following labels designate individuals in each species. Assamese macaques: ▪, crab-eating macaques: ▴, Japanese macaques: ▾, rhesus macaques: *◆*, pig-tailed macaques: grey *⚫*, and Tibetan macaques: pink *⚫*. The first PC axis accounts for most of the variation within the sample and represents overall size. The second PC axis, while depicting a much smaller amount of variation, illustrates variation in relative limb lengths and joint diameters.

**Figure 3:**
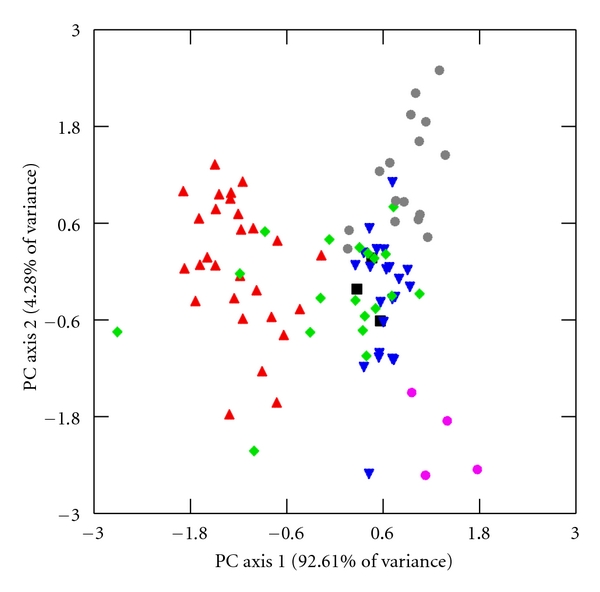
First and second PC factor scores of log-size-and-shape variables in females. The following labels designate individuals in each species. Assamese macaques: ▪, crab-eating macaques: ▴, Japanese macaques: ▾, rhesus macaques: *◆*, pig-tailed macaques: grey *⚫*, and Tibetan macaques: pink *⚫*. The first PC axis accounts for most of the variation within the sample and represents overall size. The second PC axis, while depicting a much smaller amount of variation, illustrates variation in relative limb lengths and joint diameters.

**Figure 4:**
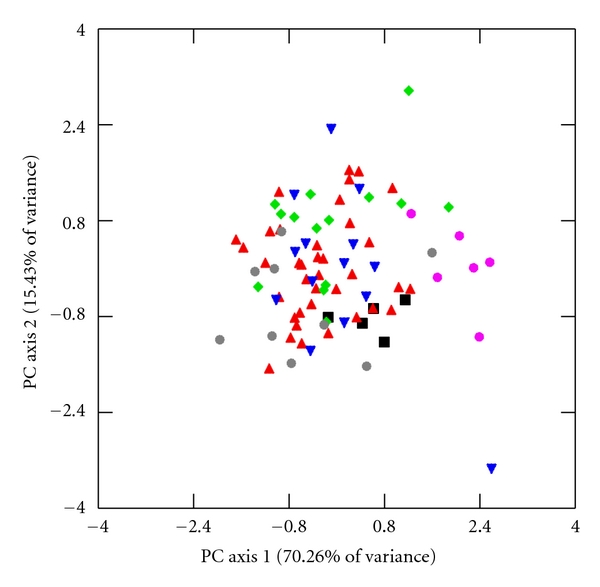
First and second PC factor scores of log-shape variables in males. The following labels designate individuals in each species. Assamese macaques: ▪, crab-eating macaques: ▴, Japanese macaques: ▾, rhesus macaques: *◆*, pig-tailed macaques: grey *⚫*, and Tibetan macaques: pink *⚫*. The first PC axis contrasts limb lengths from joint diameters while the second PC axis juxtaposes the upper limb from the lower limb.

**Figure 5:**
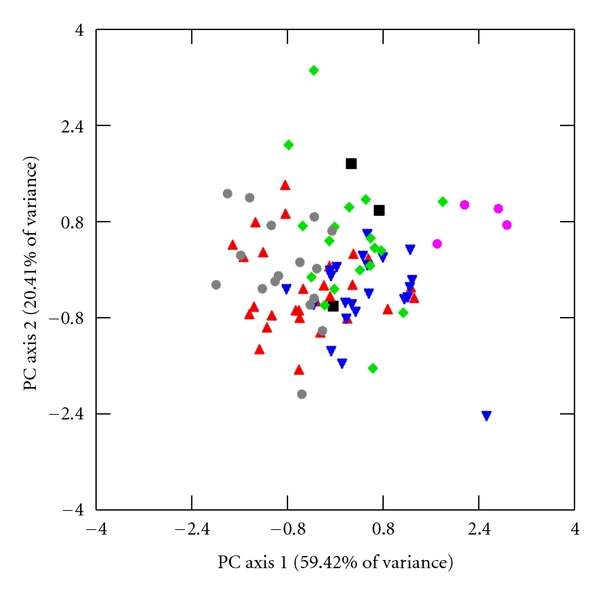
First and second PC factor scores of log-shape variables in females. The following labels designate individuals in each species. Assamese macaques: ▪, crab-eating macaques: ▴, Japanese macaques: ▾, rhesus macaques: *◆*, pig-tailed macaques: grey *⚫*, and Tibetan macaques: pink *⚫*. The first PC axis contrasts limb lengths from joint diameters while the second PC axis juxtaposes the upper limb from the lower limb.

I explore further for interspecific differences in limb lengths and joint diameters through principal component analysis (PCA) of unrotated variance-covariance matrices of HUM, RAD, FEM, TIB, HHAP, and FHAP using the log-size-and-shape and log-shape methods of Darroch and Mosimann [72]. Log-size-and-shape variables are simply each variable transformed into its natural logarithm. Log-shape variables, which capture individual variation in shape differences while controlling for differences in size, are calculated as the log-transformed value of the dependent variable subtracted from the log-transformed value of GM [72, 73]. Component loadings for the log-size-and-shape and the log-shape variables are listed in Table 9, and bivariate scatter plots of the first two factor scores of both PCAs are illustrated in Figures 2–5.

I test whether climatic factors influence interspecific variation in body size, limb lengths, and joint diameters through Spearman's rho rank-order correlation coefficient (*r_s_*) and partial correlation analyses. I use Spearman's rho rank-order correlation coefficient, a nonparametric statistical test to evaluate correlation between variables that lack normal distribution, to compare M and GM with ALT, LAT, and TMIN across all sex-specific samples (Table 10). I also use partial correlation analyses to examine the influence of ALT, LAT, and TMIN on limb lengths and joint diameters while controlling for M and GM (Table 11). As stated above, I always exclude the dependent variable from GM. Given that ambient temperatures tend to be colder with increasing altitude and latitude, it is expected that TMIN, LAT, and ALT are correlated at temperate latitudes and high elevations but act independently at lower elevations and tropical latitudes.

I transform all limb variables into their natural logarithmic values, generate all statistical tests and graphs using Systat version 11.0 [74] and PSAW Statistics 17.0 [75], and recognize statistically significant differences with a *P* value ≤0.05.

## 3. Results

### 3.1. Intra- and Interlimb Length Proportions

RAD is 0–5% shorter than HUM, and TIB is 3–10% shorter than FEM in all species (Table 4). Japanese and pig-tailed macaques have the highest intralimb length proportions of the forelimb indicating that the radius is equal or nearly equal in length to the humerus in these species while crab-eating, rhesus, Assamese, and Tibetan macaques have lower intra-forelimb proportions. In the hindlimb, members of the *fascicularis* species group (crab-eating, Japanese, and rhesus macaques) have higher intralimb length proportions than pig-tailed, Assamese, and Tibetan macaques, indicating that the length of the tibia is closer to equal to the length of the femur in the *fascicularis* species group compared with other species. 

All species share interlimb length proportions that are slightly below 1.0 indicating that forelimb length is nearly equal to that of the hindlimb (Table 4). The smallest proportioned species are from the *fascicularis* species group, and the largest proportioned are from the *sinica* species group.

### 3.2. Limb Lengths, Joint Diameters, and Overall Size

Crab-eating macaques are the lightest species based on estimations of M, followed by rhesus and Assamese macaques. Japanese and pig-tailed macaques are slightly heavier, and Tibetan macaques are the heaviest species (Table 3). Comparisons of limb lengths relative to M juxtapose small-bodied crab-eating macaques with long limbs for their size from large-bodied Tibetan macaques with short limbs (Tables 5 and 6). 

Forelimb lengths relative to GM contrast crab-eating and pig-tailed macaques with long HUM and RAD from Tibetan, Japanese, and rhesus macaques with shorter forelimbs (Tables 7 and 8). Hindlimb lengths relative to GM contrast Assamese and Tibetan macaques with short limbs from crab-eating and pig-tailed macaques with longer limbs (Tables 7 and 8). Joint diameters relative to GM juxtapose the stockier Tibetan and Japanese macaques from the pig-tailed and crab-eating macaques (Tables 7 and 8).

### 3.3. Principal Components Analysis

The first log-size-and-shape PC axis represents differences in overall size and accounts for 90.59% and 92.61% of the total sample variation in males and females, respectively (Table 9). All of the component loadings of this first axis are positive and large, and factor scores are strongly correlated with M and GM (*r* = 0.94–1.00, *P* < 0.001 for both sexes), which is to be expected given that GM is the intended size variable in this PCA [76]. Crab-eating and smaller rhesus macaques exhibit negative factor scores and are separated from the other, larger species along this axis (Figures 2 and 3). 

The second log-size-and-shape PC axis accounts for 6.51% and 4.28% of the total sample variance in males and females, respectively, (Table 9). While these percentages are much reduced compared with the first log-size-and-shape PC axis, they do illustrate subtle, yet important, variations in limb lengths and joint diameters. Component loadings along this second axis are strongest and negative for the two joint diameters and positive yet slightly weaker in the four limb lengths. This second axis does not explain variation in overall size since its factor scores are not significantly correlated with M or GM (*r* = 0.02–0.24, *P* > 0.05 for both sexes). Rather, the second log-size-and-shape PC axis contrasts HHAP and FHAP with limb lengths. Tibetan macaques, with their short limbs and large joint diameters for their large sizes, are separated along the second PC axis from pig-tailed macaques, with their long limbs, small joint diameters yet similarly large body sizes, and from crab-eating macaques, with their long limbs, small joint diameters, and much smaller body size (Figures 2 and 3). Assamese, rhesus, and Japanese macaques are intermediate in limb lengths and joint diameters compared with the larger, stockier Tibetan macaques, the larger, lankier pig-tailed macaques, and the smaller, lankier crab-eating macaques.

The component loadings of the first log-shape PC axis account for 70.26% and 59.42% of the total sample variance in males and females, respectively, (Table 9). This axis contrasts the four limb lengths with positive loadings from joint diameters with strongly negative loadings. The component loadings of the second log-shape PC axis accounts for 15.43% and 20.41% of the total sample variance in males and females and contrasts upper limb variables, which are weakly positive, from the weakly negative lower limb variables. While the first two components of the log-shape PCA accounts for a smaller amount of variation compared to the first two components of the log-size-and-shape PCA, it does illustrate interesting interspecific contrasts (Figures 4 and 5). The stockier Tibetan macaques have positive factor scores along the first log-shape PC axis. Crab-eating and pig-tailed macaques tend to overlap and fall negatively along the first log-shape PC axis despite the differences in body size in these two species. Japanese, Assamese, and rhesus macaques have intermediate factor scores between these two extremes. It should be noted that Auerbach and Sylvester [77] recently described limitations of using GM as a size factor as it can lead to a positive relationship with some of its contributing variables and a negative relationship with other contributing variables. The authors, however, demonstrate that the raw relationships between individual variables and GM in a PCA remain valid even if the sign of their coefficients might differ, which suggests that the results of these PCAs accurately describe the relationships between limb lengths and joint sizes in these samples.

### 3.4. Climate, Limbs, and Overall Size

Both GM and M are significantly positively correlated with ALT and LAT and negatively correlated with TMIN (Table 10). Partial correlation coefficients reveal moderate, yet significant, associations between limb proportions and climatic conditions while controlling for size (Table 11). While controlling for M, joint diameters are positively correlated with ALT and negatively associated with TMIN. In males, only HHAP is positively correlated with LAT, while, in females, most limb lengths and FHAP are negatively correlated with ALT and LAT and positively correlated with TMIN. When GM is held constant, HUM and RAD are negatively correlated with ALT and LAT and positively correlated with TMIN, and joint diameters are positively correlated with ALT and LAT and negatively correlated with TMIN. As noted above, altitude, latitude, and ambient temperature are interrelated and vary together. In general, as latitude and altitude increase, ambient temperature is likely to decrease, patterns that are likely to be exacerbated with fluctuations in humidity and precipitation. Progressively colder temperatures via either higher altitudes or more northern latitudes explain much of the patterns of decreasing limb lengths, increasing joint diameters, and increasing body size in these macaque species.

## 4. Discussion

Interspecific variation in overall size and limb lengths relative to body size and joint diameters suggest that *Macaca* limb morphology conforms, in part, with Bergmann's and Allen's rules. As latitude and altitude increase and average winter temperature decreases, body size and joint diameters tend to increase, and relative limb lengths tend to decrease in a pattern similar to that illustrated by Fooden [17, 19] for relative tail length variation in members of the *fascicularis* species group. Species from temperate latitudes and higher elevations, such as Tibetan and to some degree Japanese and rhesus macaques, tend to have short limbs and large joints for their size, while species from lowland tropical regions, such as crab-eating and pig-tailed macaques, tend to have long limbs. It is important to acknowledge that these climatic variables do not provide information about the adverse and additive effects of humidity and precipitation on the ability to conserve or release body heat. Thus, future work should also incorporate these climatic factors into studies of ecogeographic variation. 

Body size and proportions are one factor among many biological variables that are affected by climatic conditions in primates. Considering that macaques are unique among nonhuman primates in their geographic range within temperate latitudes and high altitudes, it is important to situate climatic adaptations in body size and proportions within this genus alongside other biological traits that vary with climatic conditions. The following discussion illustrates the importance of climatic conditions for foraging strategy, reproduction, and their relationship with body mass as well as the importance of locomotion and phylogeny in discerning interspecific variation in limb morphology in this geographically widespread genus. 

### 4.1. Climate and Seasonality in Foraging, Reproduction, and Body Size

Cold climatic conditions affect food availability, foraging strategy, and reproduction in highland and temperate macaque species. Macaques from temperate latitudes and high elevations endure seasonal stress on food resources especially during late fall through early spring when high-quality foods are limited in availability. Japanese macaques, for example, rely on nutritionally poor foods, such as tree bark, buds, and fallen seeds, during winter months, whereas they consume higher-quality foods, such as fruit and young leaves, and have greater dietary breadth during the spring, summer, and early fall [78–80]. Given that monkey troops at Yakushima, a southern location of Japanese macaques, rely on backup foods with low nutritional content during winter months [79–82], the more northern populations of this species must endure even more severe seasonal stress on food availability. The diet of Tibetan macaques at Mt. Emei, China, similarly shifts from a diverse array of leaves, fruit, fungi, insects, and food handouts from tourists during warmer months to buds, bark, and mature leaves with no food handouts during winter [60, 83]. Rhesus macaques from Northern Pakistan and Central China [53, 84] also experience fluctuations in food availability in which troops forage on a variety of leaves and flowers during the warmer months and switch to a lower-quality diet of twigs, buds, bark, and roots during winter. Rhesus macaques thrive at the edge of human settlements, and their diet, regardless of location or season, includes a substantial portion of foods produced by humans, either through crop raiding or foliage obtained in degraded forests [50–54]. It is possible that the widespread geography and extreme environmental conditions that characterize the range of rhesus macaques are partly due to their ability to thrive near human habitation, which buffers this species from the severity of seasonal fluctuations of the temperate latitudes which they inhabit. 

Macaque species that endure seasonal stress on food resources will often experience seasonal fluctuations in body weight in which body fat and muscle are used as sources of energy during scarce winter months [78]. Seasonal fluctuations in body weight characterize the northernmost troops of Japanese macaques [45, 46, 78], as well as Tibetan macaque troops at Mt. Emei, China, which lose nearly 30% of their body weight during winter [83]. Cold winter temperatures also influence activity budgets in species that dwell at temperate latitudes and higher elevations. Japanese macaques during winter, for example, forage for fallen seeds and mature leaves, food items that are evenly distributed and require little time and travel to locate [79, 85]. Despite the seasonal fluctuations in body weight in temperate latitudes and higher elevations, these monkeys remain larger in body size then their low-latitude congeners suggesting that seasonal dietary stress is not so severe as to threaten their health.

Seasonally limited diets also reduce the amount of energy available for reproduction leading to longer interbirth intervals in temperate-dwelling species compared with their tropical conspecifics and congeners. Temperate dwelling rhesus macaques, for example, tend to give birth biannually, whereas their tropical conspecifics maintain an annual birth rate [51, 55, 84]. Similarly, birthing season and female post-partum weight gain in Japanese macaques are positively correlated with environmental temperature [86].

### 4.2. Intra- and Interlimb Proportions, Phylogeny, and Locomotion

Unlike variation in overall size and limb lengths relative to size, intra-, and interlimb length proportions do not vary with climate and may reflect phylogeny, since closely related species tend to cluster together despite living in different habitats, or subtle differences in locomotor behavior, as these six species vary in the degree to which they engage in terrestrial versus arboreal quadrupedal locomotion. Given that body size is an important factor in primate positional and locomotor behavior [87], it is to be expected that differences in locomotion are factors in variation in intra- and interlimb length proportions in these species. While the extent to which locomotor behavior and phylogeny play a role in determining variation in these traits is not directly tested in this study; the following discussion sheds light on the importance of locomotion and phylogeny in limb morphology within this genus.

Assamese and Tibetan macaques of the *sinica* species group exhibit similar intra- and interlimb length proportions, which may reflect the close phylogenetic relationship between these two species as their locomotor and positional behaviors are quite different. Both species inhabit primary broadleaf evergreen forests at midelevations, up to 1900 m in Assamese and 2400 m in Tibetan macaques [59, 60]. Assamese macaques, however, are arboreal quadrupeds that rarely descend to the ground while Tibetan macaques are terrestrial quadrupeds that travel across steeply inclined mountainous slopes [59–61, 88]. 

Pig-tailed macaques have the highest limb proportions and small joint diameters for their large body sizes. As the sole representative of the *silenus* species group, they are the most distantly related species within this sample. Their long limbs and small joint diameters may represent structural adaptations to terrestrial quadrupedalism in primary forests of tropical Southeast Asia [62], an interpretation that explains their large body sizes and short tails [56, 57]. 

Members of the *fascicularis* species group share similar intra- and interlimb length proportions. Japanese macaques have the highest intralimb length proportions of both the upper and lower limb. Crab-eating and rhesus macaques exhibit nearly identical intralimb length proportions, yet rhesus macaques have markedly shorter interlimb lengths, a pattern that may reflect differences in habitat and locomotion. While the arboreal crab-eating macaques occupy more southern latitudes and the predominantly terrestrial rhesus macaques inhabit more northerly regions, both species prefer secondary degraded forests at the edge of human settlements [54]. Rhesus macaques exhibit the most varied limb proportions, which most likely result from its widespread geographic range and high degree of intraspecific genetic variation [37, 89]. Smaller rhesus monkeys have limb proportions that resemble those of crab-eating macaques, while their larger conspecifics have limb proportions that are similar to other temperate-dwelling species. The morphological similarity between the smaller rhesus and crab-eating macaques may be the result of close genetic affinities between these tropical Southeast Asian populations. Rhesus and crab-eating macaques form circumscribed hybrid zones in Northern Thailand [49]. Rhesus macaques from this region are smaller in body size and proportions and longer in relative tail length—features that suggest similar climatic adaptations or closely shared genetic history with crab-eating macaques—than their Chinese- or Indian-derived conspecifics [90]. Chinese- and Indian-derived rhesus macaques also maintain large differences in mtDNA [37, 89], which further suggest that this morphologically variable species encompasses large amounts of genetic diversity. 

Each macaque species differs in the degree that it engages in terrestrial versus arboreal quadrupedal locomotion. Arboreal crab-eating macaques, with their short limbs, small joint diameters, and small body sizes, are in contrast to the larger-bodied, terrestrial pig-tailed and Tibetan macaques, which also differ from each other in their limb proportions. Pig-tailed macaques, which travel terrestrially in lowland, primary rain forest, have long limbs and small joint diameters, while Tibetan macaques, a terrestrial species that travels across steeply inclined mountainous slopes at higher elevations, have short limbs and large joint diameters. 

Numerous studies examine primate long-bone structure to distinguish broad categories of locomotor and positional behaviors, such as to differentiate leapers, brachiators, and generalized arboreal and terrestrial quadrupeds, in extant and extinct species (e.g., [63, 91, 92]). While the data presented here do not directly compare limb morphology to identify differences in locomotion and whether or not these differences also reflect phylogenetic relationships within *Macaca*, the results from this work suggest that variations in macaque limb proportions may reflect subtle differences in the degree that each species engages in arboreal and terrestrial quadrupedalism across different substrates. Future studies of primate limb morphological variation should consider the effects of locomotion across different substrates within a single locomotor category, such as terrestrial or arboreal quadrupedal locomotion, within and among *Macaca* species or other primate genus as well as test for the effect of phylogeny in our ability to detect these interspecific variations.

## 5. Conclusions

As a genus, *Macaca* offers important insights into the significance of ecogeographic variation in primate evolution. As a geographically widespread genus with many closely related species with recent genetic divergence from one another, species within this genus are unique among nonhuman primates for inhabiting regions outside the tropics that extend into temperate- and high-altitude regions. Results of this study indicate that ecogeographic factors are important sources of variation in postcranial morphology within this genus. Climatic conditions, including ambient temperature, altitude, and latitude, influence variation in limb lengths and proportions and overall body size that function to control thermoregulation. These same climatic conditions also affect seasonality in diet, foraging strategies, and reproductive ecology within and among *Macaca* species. Although not directly tested here, this study also suggests that subtle interspecific differences in locomotion and substrate use, which also are influenced by ecological conditions, are important sources of variation in inter- and intralimb length proportions within macaques. Thus, as this study of macaques demonstrates, ecogeographic conditions should be important factors considered in explanations of the recent evolution of postcranial morphological variation in closely related primate taxa.

## Figures and Tables

**Table 1:** *Macaca* species groups.

Fooden [1]	Delson [2]	Groves [3]
*silenus-sylvanus* species group	*sylvanus *species group	*sylvanus *species group
* M. sylvanus*	* M. sylvanus*	* M. sylvanus*
* M. silenus*		
***M. nemestrina***	*silenus *species group	*M. nemestrina* species group
* M. tonkeana*	* M. silenus*	***M. nemestrina***
* M. maura*	***M. nemestrina***	* M. leonina*
* M. ochreata*	* M. tonkeana*	* M. silenus*
* M. brunnescens*	* M. maura*	* M. pagensis*
* M. hecki*	* M. ochreata*	
* M. nigrescens*	* M. brunnescens*	Sulawesi species group
* M. nigra*	* M. hecki*	
	* M. nigrescens*	*M. fascicularis *species group
*fascicularis* species group	* M. nigra*	***M. fascicularis***
***M. mulatta***		* M. arctoides*
* M. cyclopis*	*fascicularis* species group	
*** M. fuscata***	***M. mulatta***	*M. mulatta *species group
*** M. fascicularis***	* M. cyclopis*	***M. mulatta***
	*** M. fuscata***	* M. cyclopis*
*arctoides* species group	*** M. fascicularis***	***M. fuscata***
* M. arctoides*		
	*sinica* species group	*M. sinica *species group
*sinica* species group	* M. arctoides*	* M. sinica*
* M. radiata*	* M. radiata*	* M. radiata*
* M. sinica*	* M. sinica*	*** M. assamensis***
*** M. assamensis***	*** M. assamensis***	*** M. thibetana***
*** M. thibetana***	*** M. thibetana***	

Species in bold are included in this study.

**Table 2:** *Macaca* skeletons, climatic variables, and museum locations by species^1^.

Species^2^	Males	Females	ALT^3^	LAT^4^	TMIN^5^	Museum Locations^6^
A	7	4	43–1800	16.20–25.12	0.7–17.6	FMNH, MCZ
F	41	32	20–1470	−3.43–17.62	14.6–23.38	FMNH, AMNH, NMNH, MCZ, BMNH
J	17	27	23–1436	35.15–36.73	−8.4–1.9	FMNH, AMNH, NMNH, UTM
M	16	24	43–4536	−6.75–34.15	−7.6–22.6	FMNH, AMNH, NMNH, MCZ, UMUP, BMNH
N	15	19	0–1598	−0.43–10.57	9.7–25.4	FMNH, AMNH, NMNH, UMUP, BMNH
T	8	4	85–2948	26.08–32.65	−11.27–7.76	FMNH, NMNH

Total	104	110				

^1^The climatic variables encompass the geographic ranges by species for this specific sample, although the six species represented in this study inhabit regions beyond these ranges.

^2^In this table and all subsequent tables, the following abbreviations designate each species: A: Assamese macaques, F: long-tailed macaques, J = Japanese macaques, M: rhesus macaques, N: pig-tailed macaques, and T: Tibetan macaques.

^3^Altitude in m above sea level.

^4^Latitude in degrees from the Equator. Negative values indicate southern latitudes.

^5^Lowest mean monthly temperature in degrees Celsius.

^6^AMNH: American Museum of Natural History; BMNH: London Museum of Natural History; FMNH: Field Museum of Natural History; MCZ: Museum of Comparative Zoology, Harvard University; NMNH: National Museum of Natural History; UMUP: University of Philadelphia Museum of Archaeology and Anthropology; UTM: University of Tokyo Museum.

**Table 3:** Mean M (in kg) and GM ± two standard errors by species.

Species	Males		Females	
	M	GM	M	GM
A	9.39 ± 0.40	76.84 ± 1.12	6.62 ± 0.39	68.11 ± 0.78
F	5.69 ± 0.15	62.35 ± 0.55	3.82 ± 0.13	54.58 ± 0.60
J	9.79 ± 0.42	77.93 ± 1.27	7.30 ± 0.13	69.65 ± 0.36
M	9.36 ± 0.52	73.94 ± 1.63	6.55 ± 0.33	64.55 ± 1.65
N	10.90 ± 0.70	83.78 ± 2.65	7.46 ± 0.25	72.66 ± 0.87
T	14.91 ± 0.62	83.73 ± 1.50	11.59 ± 0.84	76.63 ± 1.85

**Table 4:** Log-transformed indices and the Games-Howell post hoc test results of intra- and interlimb length proportions.

Ratio	Species	*n*	Mean	SD	The Games-Howell test results
Males log(RAD/HUM)	A	5	−0.03	0.04	J > F, T, *P* < 0.05
	F	38	−0.03	0.02	
	J	14	0.00	0.04	
	M	14	−0.03	0.05	
	N	11	−0.01	0.03	
	T	6	−0.04	0.02	

Males log (TIB/FEM)	A	5	−0.06	0.04	F > N; *P* < 0.05
	F	38	−0.05	0.02	F < J; *P* = 0.01
	J	17	−0.03	0.02	T < F, J, M; N < J; *P* ≤ 0.001
	M	15	−0.05	0.03	
	N	10	−0.08	0.02	
	T	6	−0.10	0.02	

Males log (FORE/HIND)	A	5	−0.03	0.02	F < A, T; *P* < 0.05
	F	37	−0.07	0.02	J < A, T; *P* < 0.01
	J	14	−0.09	0.03	M < A, T; F < M; *P* ≤ 0.001
	M	14	−0.11	0.02	
	N	9	−0.07	0.04	
	T	6	−0.04	0.02	

Females log(RAD/HUM)	A	3	−0.05	0.01	N > A, M; *P* < 0.05
	F	28	−0.03	0.03	J > A, T; N > F; *P* < 0.01
	J	23	0.02	0.02	J > F, M, N, *P* ≤ 0.001
	M	22	−0.04	0.04	
	N	16	0.00	0.02	
	T	4	−0.04	0.02	

Females log (TIB/FEM)	A	4	−0.09	0.03	N < F; *P* < 0.01
	F	30	−0.05	0.02	N < J; *P* ≤ 0.001
	J	26	−0.04	0.02	
	M	20	−0.05	0.03	
	N	18	−0.07	0.02	
	T	4	−0.09	0.02	

Females log (FORE/HIND)	A	3	−0.04	0.01	J < T; *P* < 0.05
	F	28	−0.07	0.02	A > F; N > J; T > M; *P* < 0.01
	J	22	−0.10	0.02	A > J, M, N; F > J, M; J > M; N > M; *P* ≤ 0.001
	M	20	−0.13	0.02	
	N	16	−0.08	0.02	
	T	4	−0.04	0.02	

**Table 5:** Log-transformed indices and the Games-Howell post-hoc tests of limb lengths and joint diameters relative to M in males.

Ratio	Species	*n*	Mean	SD	The Games-Howell test results
Males log (HUM/M^0.333^)	A	5	4.33	0.04	T < A; F < N, J; *P* < 0.05
	F	40	4.30	0.05	T < F, J; M < N, *P* < 0.01
	J	16	4.30	0.04	T < N; *P* < 0.001
	M	15	4.27	0.08	
	N	10	4.39	0.07	
	T	6	4.23	0.03	

Males log (RAD/M^0.333^)	A	5	4.30	0.01	F < N; T < F; *P* < 0.05
	F	37	4.26	0.05	A > F, T; M < N; *P* < 0.01
	J	14	4.30	0.05	T < J, N; *P* < 0.001
	M	14	4.24	0.08	
	N	10	4.38	0.09	
	T	6	4.18	0.04	

Males log (FEM/M^0.333^)	A	5	4.38	0.04	N < A, M; T < A, J, M; *P* < 0.05
	F	40	4.38	0.04	T < A, F, J, M, & N; *P* ≤ 0.001
	J	17	4.40	0.06	
	M	15	4.39	0.08	
	N	11	4.49	0.08	
	T	6	4.29	0.04	

Males log (TIB/M^0.333^)	A	5	4.32	0.02	A < J; *P* < 0.05
	F	38	4.32	0.05	F < J; T < A; *P* < 0.01
	J	17	4.37	0.05	T < F, J, M, N; *P* ≤ 0.001
	M	15	4.34	0.08	
	N	10	4.40	0.10	
	T	6	4.19	0.04	

Males log (HHAP/M^0.333^)	A	5	2.20	0.02	F < N, T; M < A; *P* < 0.05
	F	40	2.10	0.05	F < A; *P* ≤ 0.001
	J	15	2.16	0.09	
	M	15	2.12	0.07	
	N	10	2.18	0.06	
	T	6	2.21	0.06	

Males log (FHAP/M^0.333^)	A	5	2.06	0.02	F < A; M < T; *P* < 0.05
	F	40	2.00	0.03	F < J, M, N, T; *P* ≤ 0.001
	J	17	2.06	0.02	
	M	15	2.04	0.02	
	N	11	2.05	0.03	
	T	6	2.08	0.02	

**Table 6:** Log-transformed indices and the Games-Howell post hoc tests of limb lengths and joint diameters relative to M in females.

Ratio	Species	*n*	Mean	SD	The Games-Howell test results
Females log (HUM/M^0.333^)	A	3	4.33	0.04	F > J, M, T; N > F; *P* ≤ 0.01
	F	28	4.31	0.04	N > J, M, T; *P* < 0.001
	J	26	4.27	0.03	
	M	23	4.25	0.07	
	N	16	4.36	0.05	
	T	4	4.23	0.03	

Females log (RAD/M^0.333^)	A	4	4.28	0.04	F > M, T; N > T; *P* ≤ 0.01
	F	28	4.28	0.04	N > F, J, M; J > M, T; *P* ≤ 0.001
	J	22	4.30	0.03	
	M	22	4.22	0.07	
	N	18	4.36	0.04	
	T	4	4.19	0.02	

Females log (FEM/M^0.333^)	A	4	4.39	0.03	A < N; T < A, F, M, *P* ≤ 0.05
	F	30	4.38	0.04	T < J; *P* < 0.01
	J	26	4.40	0.04	N > F, J, M, T, *P* ≤ 0.001
	M	21	4.38	0.08	
	N	18	4.47	0.04	
	T	4	4.30	0.03	

Females log (TIB/M^0.333^)	A	4	4.30	0.06	T < F, M < N; *P* < 0.01
	F	30	4.34	0.04	F < N; T < J, M, N; *P* ≤ 0.001
	J	26	4.36	0.04	
	M	22	4.33	0.07	
	N	18	4.40	0.04	
	T	4	4.21	0.03	

Females log (HHAP/M^0.333^)	A	3	2.09	0.07	
	F	28	2.05	0.05	T > M, N; *P* < 0.05
	J	26	2.13	0.04	F < J, T; *P* ≤ 0.001
	M	21	2.06	0.06	
	N	16	2.09	0.05	
	T	4	2.14	0.02	

Females log (FHAP/M^0.333^)	A	4	2.04	0.03	T > J, M; *P* < 0.05
	F	30	2.00	0.02	T > F; *P* < 0.01
	J	26	2.02	0.01	F < J, M, N; *P* ≤ 0.001
	M	23	2.01	0.03	
	N	18	2.03	0.02	
	T	4	2.07	0.02	

**Table 7:** Log-transformed indices and the Games-Howell post hoc test results of limb lengths and joint diameters relative to GM in males.

Ratio	Species	*n*	Mean	SD	The Games-Howell test results
Males log (HUM/GM)	A	5	0.73	0.03	J < N; *P* < 0.05
	F	37	0.73	0.03	J < F; T < N; *P* < 0.01
	J	14	0.70	0.02	T < F; *P* = 0.001
	M	14	0.70	0.03	
	N	9	0.74	0.02	
	T	6	0.70	0.01	

Males log (RAD/GM)	A	5	0.70	0.01	M < N; *P* = 0.05
	F	37	0.70	0.03	T < A, F. J; *P* ≤ 0.01
	J	14	0.70	0.02	T < N; *P* = 0.001
	M	14	0.68	0.04	
	N	9	0.72	0.03	
	T	6	0.65	0.02	

Males log (FEM/GM)	A	5	0.78	0.04	T < F, N; *P* < 0.01
	F	37	0.82	0.02	T < M; *P* = 0.001
	J	14	0.80	0.04	
	M	14	0.83	0.03	
	N	9	0.84	0.04	
	T	6	0.76	0.02	

Males log (TIB/GM)	A	5	0.72	0.01	A < F, T; T < N; *P* < 0.01
	F	37	0.76	0.02	A < J, M; T < F, J, M; *P* ≤ 0.001
	J	14	0.78	0.03	
	M	14	0.77	0.03	
	N	9	0.76	0.05	
	T	6	0.66	0.02	

Males Log (HHAP/GM)	A	5	−1.40	0.03	A > F, M; *P* < 0.05
	F	37	−1.46	0.05	T > F, J, N; *P* < 0.01
	J	14	−1.44	0.08	T > M; *P* < 0.001
	M	14	−1.46	0.05	
	N	9	−1.47	0.05	
	T	6	−1.32	0.04	

Males log (FHAP/GM)	A	5	−1.54	0.03	T > A, M; *P* ≤ 0.01
	F	37	−1.55	0.04	T > F, J, N; *P* ≤ 0.001
	J	14	−1.54	0.04	
	M	14	−1.53	0.06	
	N	9	−1.59	0.06	
	T	6	−1.45	0.02	

**Table 8:** Log-transformed indices and the Games-Howell post hoc test results of limb lengths and joint diameters relative to GM in females.

Ratio	Species	*n*	Mean	SD	The Games-Howell test results
Females log (HUM/GM)	A	3	0.76	0.01	M > J; *P* < 0.05
	F	28	0.76	0.02	A > J, M; N > M; *P* < 0.01
	J	22	0.69	0.02	F > J, M; N > J; *P* ≤ 0.001
	M	19	0.71	0.02	
	N	16	0.75	0.02	
	T	4	0.71	0.02	

Females log (RAD/GM)	A	3	0.71	0.01	T < F, J; J < N; *P* ≤ 0.01
	F	28	0.72	0.02	M < F, J, N; T < N; *P* < 0.001
	J	22	0.72	0.02	
	M	19	0.67	0.02	
	N	16	0.74	0.02	
	T	4	0.67	0.01	

Females log (FEM/GM)	A	3	0.82	0.01	T < F, J; J < M; *P* < 0.05
	F	28	0.83	0.02	F < N; T < M, N; *P* ≤ 0.01
	J	22	0.82	0.03	J < N; *P* < 0.001
	M	19	0.84	0.02	
	N	16	0.85	0.02	
	T	4	0.78	0.02	

Females log (TIB/GM)	A	3	0.73	0.02	T < F, J, M, N; *P* < 0.01
	F	28	0.78	0.02	
	J	22	0.78	0.02	
	M	19	0.79	0.02	
	N	16	0.78	0.02	
	T	4	0.68	0.02	

Females log (HHAP/GM)	A	3	−1.49	0.03	T < A, J; *P* < 0.05
	F	28	−1.50	0.05	F < J; *P* < 0.01
	J	22	−1.45	0.05	N < J; *P* < 0.001
	M	19	−1.49	0.06	T > F, M, N; *P* ≤ 0.001
	N	16	−1.53	0.04	
	T	4	−1.38	0.02	

Females log (FHAP/GM))	A	3	−1.53	0.05	J > F, N; *P* < 0.05
	F	28	−1.59	0.04	M > F, N; *P* < 0.01
	J	22	−1.56	0.03	T > F, J. M; *P* ≤ 0.01
	M	19	−1.54	0.05	T > N; *P* = 0.001
	N	16	−1.59	0.03	
	T	4	−1.46	0.03	

**Table 9:** PCA component loadings.

	Log-size-and-shape				Log-shape			
	Males		Females		Males		Females	
	Axis 1	Axis 2	Axis 1	Axis 2	Axis 1	Axis 2	Axis 1	Axis 2
FHAP	0.144	−0.040	0.146	−0.030	−0.043	−0.003	−0.033	−0.035
HHAP	0.156	−0.056	0.153	−0.047	−0.062	0.024	−0.052	0.026
RAD	0.133	0.025	0.135	0.024	0.022	0.011	0.021	0.008
HUM	0.125	0.020	0.121	0.021	0.019	0.008	0.023	0.000
FEM	0.131	0.032	0.135	0.024	0.029	−0.006	0.021	−0.001
TIB	0.124	0.036	0.128	0.021	0.035	−0.004	0.021	0.002

% of total variance	90.59	6.51	92.61	4.28	70.26	15.43	59.42	20.41

**Table 10:** Spearman's rank order correlation coefficient (*r_s_*) of M and GM with climate variables.

	Males				Females			
	M		GM		M		GM	
	*r_s_*	*P*	*r_s_*	*P*	*r_s_*	*P*	*r_s_*	*P*
ALT	0.444	<0.001	0.384	0.001	0.454	<0.001	0.280	0.012
LAT	0.545	<0.001	0.520	<0.001	0.489	<0.001	0.341	0.002
TMIN	−0.667	<0.001	−0.629	<0.001	−0.449	<0.001	−0.320	0.004

**Table 11:** Partial correlation coefficients of climate and limb variables controlling for GM and M.

			Males		Females	
Control variable	Climate variable	Limb variable	Partial *r*	*P*	Partial *r*	*P*
M	ALT	HUM	−0.031	—	−0.457	<0.001
		RAD	−0.123	—	−0.460	<0.001
		FEM	−0.015	—	−0.460	<0.001
		TIB	−0.051	—	−0.381	<0.001
		HHAP	0.226	0.04	0.031	—
		FHAP	0.319	0.004	−0.193	—

	LAT	HUM	−0.068	—	−0.480	<0.001
		RAD	−0.010	—	−0.362	0.001
		FEM	0.011	—	−0.300	0.004
		TIB	0.157	—	−0.201	—
		HHAP	0.203	—	0.068	—
		FHAP	0.398	<0.001	−0.208	0.05

	TMIN	HUM	−0.022	—	0.449	<0.001
		RAD	−0.038	—	0.362	0.001
		FEM	−0.078	—	0.287	0.006
		TIB	−0.179	—	0.197	—
		HHAP	−0.275	0.01	−0.123	—
		FHAP	−0.398	<0.001	0.245	0.019

GM	ALT	HUM	−0.236	0.04	−0.410	<0.001
		RAD	−0.487	<0.001	−0.466	<0.001
		FEM	−0.195	—	−0.444	<0.001
		TIB	−0.321	0.006	−0.300	0.007
		HHAP	0.276	0.02	0.417	<0.001
		FHAP	0.425	<0.001	0.503	<0.001

	LAT	HUM	−0.538	<0.001	−0.635	<0.001
		RAD	−0.361	0.002	−0.331	0.003
		FEM	−0.325	0.005	−0.367	0.001
		TIB	−0.033	—	−0.032	—
		HHAP	0.227	0.05	0.361	0.001
		FHAP	0.465	<0.001	0.412	<0.001

	TMIN	HUM	0.445	<0.001	0.631	<0.001
		RAD	0.379	0.001	0.366	0.001
		FEM	0.301	0.01	0.390	<0.001
		TIB	0.102	—	0.058	—
		HHAP	−0.266	0.02	−0.412	<0.001
		FHAP	−0.407	<0.001	−0.404	<0.001
